# Bioethanol in Biofuels Checked by an Amperometric Organic Phase Enzyme Electrode (OPEE) Working in “Substrate Antagonism” Format

**DOI:** 10.3390/s16091355

**Published:** 2016-08-25

**Authors:** Mauro Tomassetti, Gabriele Spuri Capesciotti, Riccardo Angeloni, Elisabetta Martini, Luigi Campanella

**Affiliations:** Department of Chemistry, University of Rome “Sapienza”, P.le A. Moro 5, Rome 00185, Italy; gcapesciotti@gmail.com (G.S.C.); angeloni.698028@studenti.uniroma1.it (R.A.); elisabettamartini@libero.it (E.M.); luigi.campanella@uniroma1.it (L.C.)

**Keywords:** catalase OPEE, biofuels, bioethanol, determination

## Abstract

The bioethanol content of two samples of biofuels was determined directly, after simple dilution in decane, by means of an amperometric catalase enzyme biosensor working in the organic phase, based on substrate antagonisms format. The results were good from the point of view of accuracy, and satisfactory for what concerns the recovery test by the standard addition method. Limit of detection (LOD) was on the order of 2.5 × 10^−5^ M.

## 1. Introduction

### 1.1. Biosensors

Although in recent years separative methods have assumed huge importance in analytical chemistry, their typical application nevertheless needs a relatively long time and has considerable costs. Analytical chemistry is therefore constantly looking for methods and techniques able to get good results more quickly, but at the same time being precise and accurate.

Electrochemical sensors and biosensors are especially suitable for the solution of many analytical problems of various kinds, concerning different matrices. Currently they are applied in increasing numbers of various scientific and technological fields [1,2,3,4].

These devices allow the performance of rapid and inexpensive determinations of analytical interest; for instance, monitoring biological or vegetable matrices. In particular, highlighting how specific “chemical indicators” correlate to the preservation and degradation state of foods, or to human health conditions [5,6,7]. 

Biosensor technology in particular has achieved several goals as a method for the analysis and control of biological and chemical systems; however, it can also find applications in other fields, such as agro-food, environmental monitoring systems, and in microbial contamination, pharmaceutical analysis, and drug control [6,7,8,9,10].

### 1.2. Organic Phase Biosensors

Until a few years ago, however, applying these devices to the analysis of highly hydrophobic substances insoluble or poorly soluble in aqueous solutions was rather difficult. In recent times, therefore, special enzymatic biosensors, so-called OPEEs (Organic Phase Enzyme Electrodes) have been developed, able to directly perform in organic solvents. These set up a new class of biosensors which can be used in all cases in which insoluble or poorly soluble matrices in aqueous media must be quickly analysed [10,11].

All of this has greatly expanded the applications of biosensors, making the biosensor technique one of the most versatile in the analytical field. Its use has also expanded to the control of lipophilic biological molecules and to more productive or industrial sectors, such as those of petroleum product control and hydrophobic pesticides analysis [12,13].

Precise biosensor methods, using OPEEs, can find wide application in the biofuels field. They have come to the forefront in recent years, both for the chronic environmental problems, and for the instability of oil prices that destabilizes the energy sector [14].

### 1.3. Biofuels

Biofuels are real and proper agricultural products, but are able to replace (at least in part) fuel derived from petroleum. It is estimated that their use would allow a 70% reduction in greenhouse gas emissions from private transport and reduce the import of petroleum from countries that have a monopoly. These arguments were evaluated seriously by the European Union, which has imposed the aim to satisfy at least 2% of the national demand for energy with the use of biofuels to all member countries. 

Bioethanol today is certainly the most interesting biofuel, its worldwide production being estimated at between 11 and 11.5 million tons/year (of which more than 90% is in the US and Brazil). Its use already contributes to a beneficial reduction in emissions of carbon monoxide, aromatic hydrocarbons, and more than 70% of sulphur dioxide [15,16,17,18]. Bioethanol can be mixed directly into petrol in varying proportions, depending on local regulations regarding fuel: 20% or more in Brazil, 5.7%–10% in the United States, 5% in Europe, to mention just the most important areas involved [18,19,20].

### 1.4. Aim of the Research

Methods for rapid determination of ethanol content in green fuels and biofuels are therefore increasingly in demand. Our research group has long experience in the development of sensors, biosensors and immunosensors of different types [2,6,21,22], but in particular in fabricating OPEEs [23,24,25,26,27,28,29] and Organic Phase Immuno Electrodes (OPIEs) [30,31]. One of the most recent OPEEs we developed for the determination of ethanol in organic solvents [32] is based on the catalase enzyme. In a previous article, this device was applied to determine ethanol in green gasolines [33], which generally contain very low concentrations of ethanol, on the order of 0.003%–0.01% by volume. In this paper, we have instead used the same biosensor, operating in decane, for the determination of bioethanol content in real commercial biofuels. The concentration of bioethanol, as already said, is much higher, in our case on the order of 10% by volume.

## 2. Experimental Section

### 2.1. Materials and Apparatus

Anhydrous ethanol and methanol, monobasic potassium phosphate, anhydrous dibasic phosphate, all of analytical grade, were supplied by Carlo Erba (Milan, Italy); tert-butylhydroperoxide in decane (5.5 M), catalase (E.C. 1.11.1.6) from bovine liver (21,000 Umg^−1^), D-9777 dialysis membrane, were supplied by Sigma Aldrich (Milan, Italy); decane, propanol, isopropanol, butanol, tert-butanol, methyl-tert-butyl-ether (MTBE), potassium chloride, kappa-carrageenan, all RPE, were supplied by Fluka (Buchs, Switzerland); The titre of all the solutions in organic solvent, as well as the standard ethanol solutions used, was checked using GC–MS, as described in previous paper [32]. Measurements by the biosensor were performed using the following apparatus: an amperometric electrode “Model 4000-1” for the oxygen (Clark type), with the external cap replaced by a cap made of polytetrafluoroethylene (PTFE), coupled with a “Biosensor Amperometric Detector” potentiostat, Model 3001 ABD, both supplied by Universal Sensor Inc. (New Orleans, LA, USA) and connected with an Amel (Milan, Italy) model 868 analog recorder. Lastly, a magnetic stirrer, model F20ST, was supplied by Falc. Instrument (Lurano, Italy). Analytical balance was supplied by Mettler AE 420 (Milan, Italy); 25, 50, 100, 200, 500, 1000 µL micropipettes were supplied by Gilson (Cinisello Balsamo, Milan, Italy/Middleton, WI, USA).

### 2.2. Samples

Two samples of biofuel containing bioethanol were supplied by a company located in the northeast of Italy. This company produces bioethanol to be used as biofuel, added to ordinary hydrocarbons that constitute unleaded petrol fuel. Each analysed sample contained 10% by volume bioethanol (nominal value provided by the producing company).

## 3. Methods

### Biosensor Fabrication and Measurements

**Figure 1:**
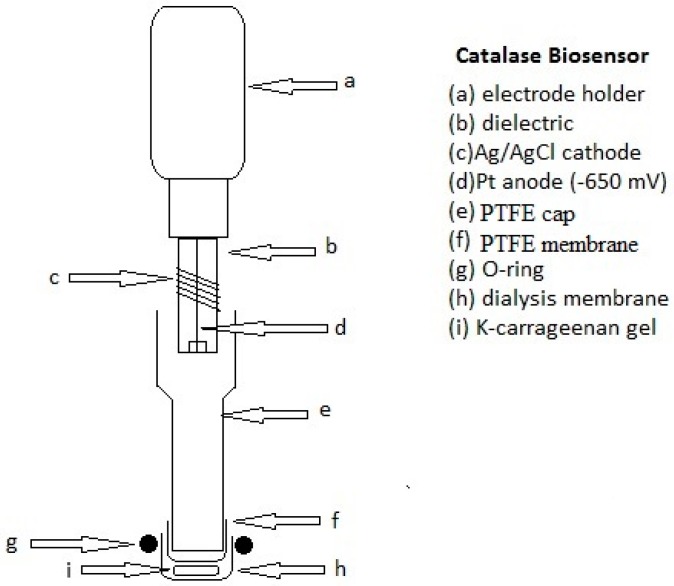
Catalase biosensor assembly.

A kappa-carrageenan disk containing the immobilized enzyme was placed at the PTFE electrode tip, sandwiched between a gas-permeable (PTFE) membrane and a dialysis membrane (Figure 1). 

Since the plastic cap of the commercially-available electrode was not able to operate in organic solvents (as it was quickly attacked), it was replaced with a similar PTFE cap of the same size. Additionally, the original rubber O-ring used to fix the gas-permeable membrane was replaced by a similar PTFE ring. The catalase enzyme was immobilized in a small kappa-carrageenan gel disk, prepared as described in detail in previous papers [32,33]. Measurements were carried out in a glass cell, thermostated at 23 °C in open air, containing 10 mL of decane solution under gentle magnetic stirring, in which the biosensor was dipped. 

The catalase biosensor and the substrate (tert-butylhydroperoxide) in decane—into which the enzyme competitive format takes place—function as follows: in the first reaction (1), the immobilized catalase enzyme catalyses (in decane, RH) an oxidation reaction in the presence of the hydroperoxide [32], which produces oxygenated species deriving from decane oxidation [33,34]. In this reaction, consumption of the dissolved oxygen occurs.

(1) $$\text{t-buOOH}+O_{2}+2\mathrm{RH}\overset{catalase}{⟶}\text{t-buOH}+2\mathrm{RO}+H_{2}O$$

In the second reaction (2), the catalase enzyme catalyses a reaction in which the hydroperoxide oxidizes the analyte to be determined (i.e., the ethanol) to acetaldehyde. In this reaction, no change in dissolved oxygen concentration occurs.

(2) $$\text{t-buOOH}+CH_{3}CH_{2}\mathrm{OH}\overset{catalase}{⟶}\text{t-buOH}+CH_{3}\mathrm{CHO}+H_{2}O$$

**Figure 2:**
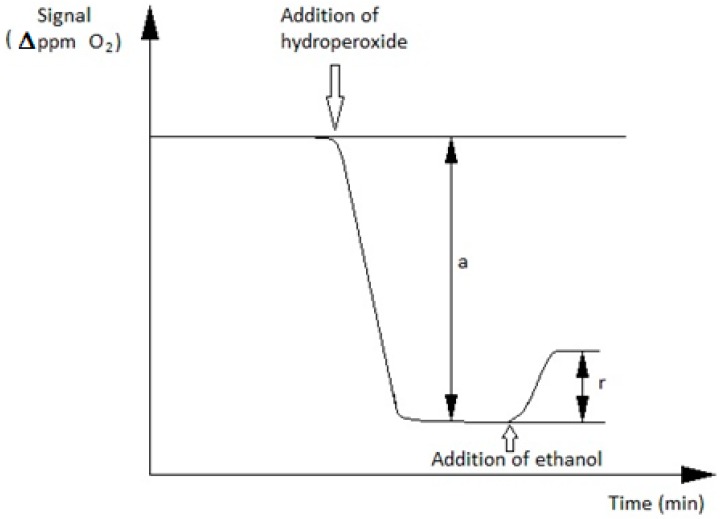
Trend of catalase biosensor response during a typical measurement of ethanol concentration, operating in decane.

This causes a partial restoration of the initial O_2_ concentration in the decane solution, when ethanol is also added, since the second reaction competes with the first one for the t-buOOH substrate (Figure 2). 

The extent of this oxygen “restoration” is measured by the Clark oxygen electrode, and can be linked to the concentration of the ethanol contained in the decane solution. This allows a suitable calibration straight line to be obtained, by plotting the “r/a” ratio (see Figure 2) vs. the increasing final total concentrations of the added ethanol in the reaction cell solution. The construction of a typical calibration curve, recorded under these conditions, was described in detail in a previous paper [33].

## 4. Results and Discussion

### 4.1. Choice of the Solvent

First of all, we tried to evaluate the choice of decane as organic solvent for the two enzymatic reactions on which the method is based. Decane has a low dielectric constant (є = 2.00) and high hydrophobicity (i.e., log *p* = 5.802), that (as is well known) makes it extremely advantageous when used as solvent for measurements by OPEEs [35,36,37] or OPIEs [31,38]. In fact, on the basis of these characteristics, decane guarantees that an enzyme is not rapidly denatured in this solvent, as often occurs in the case of several other more polar organic solvents; on the contrary, enzymatic activity remains high and persistent for a long time [31,35,36,37,38]. 

We also considered the functioning of the OPEE enzymatic measurement in decane based on the I.S.F (Iwuoha and Smith function) algorithm [39,40], which gives an indicative value of the diffusivity of the substrate to the enzyme in the solution used for the measurement. In fact, if the function of Iwuoha, Smith, and Lyons [39,40] is applied 1/ηε, (where η is the absolute viscosity of the solvent and ε is its dielectric constant) to the enzymatic reaction of the method under test, the effect of the solvent used on the diffusion of the substrate to the enzymatic membrane is evidenced. This effect can be easily evaluated [41,42], as the diffusion is actually more favourable when the enzyme reaction takes place in an organic solvent such as decane, rather than in aqueous solution. Indeed, according to results reported in the literature, the higher the value of this function (that is, the lower the value η of the viscosity and/or of the dielectric constant ε of the solvent), the greater the diffusion of the analyte from the solvent to the enzymatic membrane [39,40,41,42]. However, the viscosity and dielectric constant values are very low for an organic solvent such as decane (at room temperature, η = 0.92 mPa·s; ε = 2.00) when compared to the corresponding values for water (η = 0.89 mPa·s; ε = 78.4). Therefore, the diffusion process is more favourable in decane than in water. 

On the other hand, it is well known that the sensitivity of an enzymatic reaction, working in organic media, depends on the deviation from Michaelis–Menten kinetic of the response of the enzyme (catalase in our case) to the substrate molecule (i.e., ethanol herein). It can be determined from the “Hill coefficient”, “x” of the following equation [43,44,45]:

log Y/(1 − Y) = x log (ΔI/I_50_) (3)

where ΔI is the change in current caused by the enzymatic reaction for a given concentration of ethanol, and I_50_ is the current achieved when the progress of the enzymatic reaction has reached 50%. The Hill coefficient “x” is an empirical parameter introduced to consider the cooperative effects in the non-Michaelis–Menten kinetics description. This equation accounts for allosteric binding at sites other than the active site. Generally, when “x” (the “Hill coefficient”) is <1, there is negative cooperativity; when x = 1, there is no cooperativity; and when x > 1, there is positive cooperativity.

**Figure 3:**
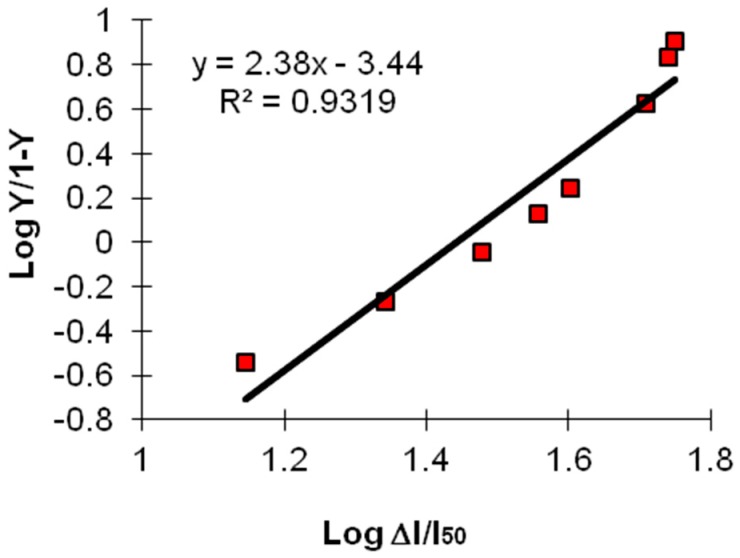
Hill’s coefficient “x” (in decane, for ethanol determination with catalase enzyme) using equation Log (Y/1 − Y) = x log (ΔI/I_50_); in all cases, Relative Standard Deviation % (RSD%) ≤ 5.5. Concerning the (x) slope, as the slope value is greater than 1, higher is the sensitivity of biosensor.

The Hill coefficient (x) is generally found to be greater than 1 (equal to or very close to 2) if the enzymatic reaction takes place in a good lipophilic organic solvent, whereas, if the reaction takes place in aqueous solution, amounts reach maximum at about 1.5. This should mean [39,46] that the biosensor is more sensitive if the enzymatic reaction takes place in a lipophilic organic solvent, in which the deviation from unit value is greater than in aqueous solvent [46]. As can be observed in Figure 3, in which the Hill equation was experimentally applied to our OPEE, responsive to ethanol, working in decane, the coefficient “x” value is actually very close to about 2. This confirms the correct choice of decane as organic solvent for our OPEE [43,44].

### 4.2. Analytical Results

**Figure 4:**
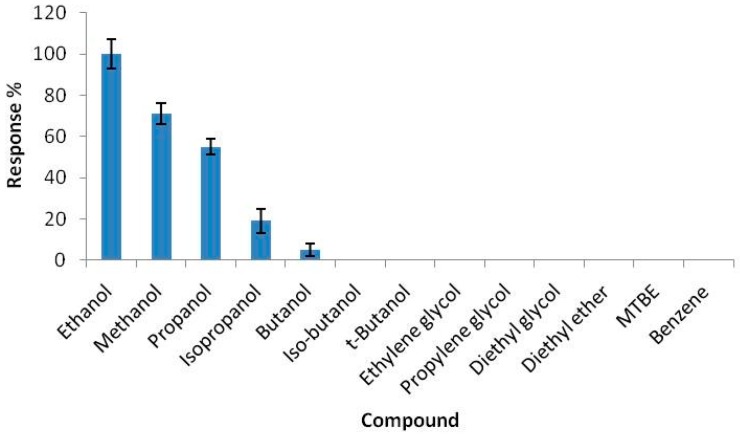
Selectivity of ethanol biosensor towards possible more-common alcohol interferents.

The catalase OPEE working in decane was also optimized from the analytical point of view in previous papers [32,33]; however, for easy availability for the reader, the main analytical data are collected in Table 1 and Table 2, while the selectivity is illustrated as histograms in Figure 4. 

It is noted the response of the biosensor decreases rapidly with increasing chain length and the complexity of the alcohol molecule. The biosensor also responds to methanol (an alcoholic molecule smaller than that of ethanol), but its response in this case is lower (about 30%) compared to that of ethanol. Taking equal to 100% the response to ethanol.

The reported data show that the catalase enzyme biosensor shows good precision values, satisfactory lifetime, and good selectivity, working in decane. It was therefore used for the control of ethanol content in two different samples of biofuel containing bioethanol up to 10% by volume (nominal value provided by the producer firm).

To this end, repeated measurements were carried out with catalase OPEE on these samples. Since the response of the biosensor is linear for concentration range 5 × 10^−5^–5 × 10^−4^ M, the samples were diluted 150 times by volume with decane before the measurement, so their ethanol concentration was in the linearity range of the method. On the other hand, the dilution procedure with decane also allows the volatility of the biofuels to be lowered, thus reducing the possible experimental errors associated with volumetric sampling of biofuel caused by possible evaporation. The results obtained are illustrated in Table 3. In Table 3 the experimental values of three determinations are reported, performed on each of the two diluted samples of biofuel, expressed as percent by volume of ethanol, g/L, or as moles/litre (i.e., M). Accounting for dilution, the mean of the three tests is compared with the nominal value provided by the manufacturer. 

The *t*-test was also applied, resulting as “not significant” in both cases. This of course increases our confidence in the performed measurements. However, even the values of recoveries, using the standard addition method reported in Table 4, show in all cases an agreement within 80%–94%, not quite excellent, but certainly satisfactory.

On the other hand, the precision of the method is certainly good: RSD% data in Table 3, in each case, range between 6.2% and 6.4% values.

## 5. Conclusions

It can therefore be concluded that the proposed catalase OPEE is perfectly able to be used for direct measure of the percentage of bioethanol contained in biofuels. Any pre-treatment that is not a simple dilution in decane is necessary. All this is possible because the proposed biosensor is suitable for working in an organic solvent, such as decane, where gasoline is easily soluble. On the other hand, decane is a particularly suitable organic solvent to be used as a medium in enzymatic biosensors [39,40,46]. It is widely explained in the discussion section of the present paper, according to the Hill’s equation results. It is also interesting to remark that for the first time it was demonstrated how it was possible to apply this equation to the case of an OPEE working in a “substrate antagonism format”.

## Figures and Tables

**Table 1:** Principal analytical data referring to the catalase organic phase enzyme electrode (OPEE) and equation of calibration straight line for ethanol, operating in decane and using t-BuOOH.

Hydroperoxide Used as Substrate	t-Butylhydroperoxide
Equation of calibration curve to ethanol	Y = 143.0 (±4.8)X + 8.0 (±1.1)
Linearity range (mM)	4.2 × 10^−2^ − 3.7 × 10^−1^
Limit of detection (LOD) (mM)	0.02
Pooled SD	≤8
Relative Standard Deviation % (RSD%) (*n* ≥ 5)	1.2

**Table 2:** Biosensor response time and lifetime using t-BuOOH, operating in decane.

Hydroperoxide Used for Ethanol Measurements	Biosensor Lifetime (Days)	Mean Response Time vs. Ethanol (*n* ≥ 5) (min)	Mean Response Time vs. Hydroperoxide (*n* ≥ 5) (min)	Total Response Time (*n* ≥ 5) (min)
Tert-butyl-hydroperoxide	≥7	10 (±5)	5 (±3)	15 (±8)

**Table 3:** Analysis of bioethanol in two different biofuel samples using catalase OPEE.

	Measure	Ethanol Concentration (M)	Ethanol Concentration as (g/L) d = 790 (g/L) MW = 46.07	Ethanol Concentration as (*v*/*v*)%	*t*-Test: Two Sided, ν A = ν B = 3 − 1 = 2, *p* = 95%		
Nominal value of Ethanol in biofuel (sample A) after being diluted 150 times by volume		1.14 × 10^−2^	5.25 × 10^−1^	6.6 × 10^−2^	t exp.	t critic	Result of the test
Found ethanol in biofuel (A) (diluted 150 times by volume), using biosensor	1	9.27 × 10^−3^	4.27 × 10^−1^	5.41 × 10^−2^	−4.210	4.303	Not significant
	2	1.05 × 10^−2^	4.84 × 10^−1^	6.12 × 10^−2^			
	3	9.94 × 10^−3^	4.58 × 10^−1^	5.80 × 10^−2^			
	Mean	9.90 × 10^−3^	4.56 × 10^−1^	5.78 × 10^−2^			
	SD	6.16 × 10^−4^	2.84 × 10^−2^	3.59 × 10^−3^			
	RSD%	6.22	6.22	6.22			
Nominal value of Ethanol in biofuel (sample B) after being diluted 150 times by volume		1.14 × 10^−2^	5.25 × 10^−1^	6.6 × 10^−2^	t exp.	t critic	Result of the test
Found ethanol in biofuel (A) (diluted 150 times by volume), using biosensor	1	9.59 × 10^−3^	4.42 × 10^−1^	5.59 × 10^−2^	−3.723	4.303	Not significant
	2	9.73 × 10^−3^	4.48 × 10^−1^	5.67 × 10^−2^			
	3	1.08 × 10^−2^	4.96 × 10^−1^	6.27 × 10^−2^			
	Mean	1.00 × 10^−2^	4.62 × 10^−1^	5.85 × 10^−2^			
	SD	6.39 × 10^−4^	2.94 × 10^−2^	3.73 × 10^−3^			
	RSD%	6.37	6.37	6.37			

**Table 4:** Recovery tests by standard addition method.

Ethanol Concentration Determined after 150 Times Dilution by Volume (M) (*n* = 5); RSD% ≤ 5		Ethanol Concentration Added (M)	Total Ethanol Concentration Found (M) (*n* = 5); RSD% ≤ 5	Recovery %
Nominal value after dilution (M)	1.00 × 10^−2^	0.50 × 10^−2^	1.25 × 10^−2^	80.0
Nominal value after dilution (M)	1.00 × 10^−2^	0.50 × 10^−2^	1.42 × 10^−2^	94.0
